# More Than a Gut Feeling—A Combination of Physiologically Driven Dissolution and Pharmacokinetic Modeling as a Tool for Understanding Human Gastric Motility

**DOI:** 10.1021/acs.molpharmaceut.4c00117

**Published:** 2024-07-03

**Authors:** Michał Romański, Marcela Staniszewska, Justyna Dobosz, Daria Myslitska, Jadwiga Paszkowska, Bartosz Kołodziej, Svitlana Romanova, Grzegorz Banach, Grzegorz Garbacz, Inese Sarcevica, Yeamin Huh, Vivek Purohit, Mark McAllister, Suet M. Wong, Dorota Danielak

**Affiliations:** † Department of Physical Pharmacy and Pharmacokinetics, Poznan University of Medical Sciences, 3 Rokietnicka St., 60-806 Poznań, Poland; ‡ Physiolution Polska, 74 Piłsudskiego St., 50-020 Wrocław, Poland; § Worldwide Research and Development, Pfizer R&D UK Ltd., Sandwich, CT13 9NJ, U.K.; ∥ Worldwide Research and Development, Pfizer Inc., Groton, Connecticut 06340, United States

**Keywords:** biopredictive dissolution testing, capsules, gastrointestinal tract, *in vitro*–*in vivo* modeling, pharmacokinetics, PhysioCell

## Abstract

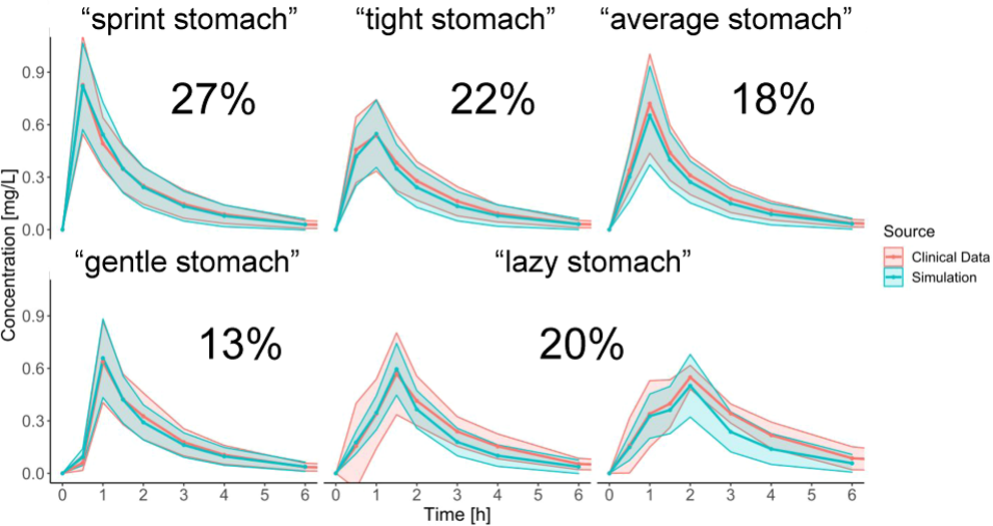

*In vivo* studies of formulation performance with *in vitro* and/or *in silico* simulations are often limited by significant gaps in our knowledge of the interaction between administered dosage forms and the human gastrointestinal tract. This work presents a novel approach for the investigation of gastric motility influence on dosage form performance, by combining biopredictive dissolution tests in an innovative *PhysioCell* apparatus with mechanistic physiology-based pharmacokinetic modeling. The methodology was based on the pharmacokinetic data from a large (*n* = 118) cohort of healthy volunteers who ingested a capsule containing a highly soluble and rapidly absorbed drug under fasted conditions. The developed dissolution tests included biorelevant media, varied fluid flows, and mechanical stress events of physiological timing and intensity. The dissolution results were used as inputs for pharmacokinetic modeling that led to the deduction of five patterns of gastric motility and their prevalence in the studied population. As these patterns significantly influenced the observed pharmacokinetic profiles, the proposed methodology is potentially useful to other *in vitro*–*in vivo* predictions involving immediate-release oral dosage forms.

## Introduction

With advances in computational power and the availability of modeling and simulation to support pharmaceutical development processes, it appears to be reasonable to ask how long we will need to rely upon dosing formulations to healthy volunteers in relative bioavailability or bioequivalence studies to confirm the impact of changes to formulation design on *in vivo* performance. The fact is we are now on the way to replacing an appreciable part of *in vivo* pharmacokinetic (PK) studies with *in vitro* experiments and/or *in silico* simulations.^1−3^ Computational modeling and simulation implemented by industry, academia, and regulators improved the rationality and productivity of new drug product development, reduced the number of humans participating in clinical trials, helped informed decision-making, and lowered the costs of new medicines.^1,2^ The approach has recently gained the formal term “Model-Informed Drug Development” and has become a standard in all stages of medicinal product development.^2−4^ Currently, the US Food and Drug Administration (FDA) and European Medicines Agency (EMA) have signaled acceptance and encouraged the use of mechanistic physiologically based pharmacokinetic (PBPK) modeling and simulations in particular tasks within regulatory submissions. These include assessing the clinical relevance of drug–drug interactions or drug product’s quality attributes, and supporting dose recommendations in special populations, e.g., pediatric or hepatic-impaired.^5,6^

Over the past decade, physiologically based biopharmaceutics modeling (PBBM) has evolved from PBPK to focus more on understanding drug release, dissolution, and absorption. The principal role of PBBM is to provide a mechanistic and quantitative linking between the *in vitro* measurable properties of drug substance/drug product and human exposure to the particular product batch.^7−9^ This, in turn, helps establish clinically relevant drug product specifications, which are the foundation of the modern medicinal product development paradigms—Quality by Design and patient-centric drug product quality standards.^7,8^ Ultimately, PBPK and PBBM reduce the need to conduct human studies in which participants receive no medical benefit. One of the most evident examples is enabling virtual bioequivalence trials in generic product applications.^7−9^ However, experimental limitations and knowledge gaps about the behavior of active pharmaceutical ingredients (API) and their drug products in patients prevent a complete replacement of clinical trials with computer-aided predictions. The greatest challenge is reflecting the variance of human physiology within the population (intersubject variability) as well as in the same individuals over time (intrasubject variability) through a finite number of *in vitro* experiments or reliable input data for computer simulations.^7−9^

The variability in the PK of drugs after oral administration arises from three main sources: the properties of the API itself, formulation, and physiology of the body. These three properties dictate dosage form disintegration, drug substance dissolution, and absorption. On the other hand, API physicochemical properties and physiology contribute to the variability of drug distribution from blood to the target tissues and undesired sites, metabolism, and excretion.^7−11^ For solid oral dosage forms, which are the most commonly used medicinal products worldwide, the intersubject and intrasubject variability of the human gastrointestinal tract (GIT) plays a crucial role in determining *in vivo* drug product performance. A significant source of variability is associated with gastric motility, which encompasses a range of physiological variables including the rate of fluid flow from the stomach to the duodenum, intragastric mechanical agitation, and the time of complete rapid gastric emptying due to intense muscle contractions (“housekeeper wave”).^10,11^ The aforementioned processes have the utmost importance for mechanically stress-sensitive immediate-release (IR) forms containing API that undergo rapid dissolution in gastric juice and rapid permeation from the small intestine to the blood. Under such conditions, gastric motility becomes a limiting factor for drug absorption. Therefore, the variance in plasma drug concentration profiles in the population may mirror gastric motility variability.^11,12^

In this work, we hypothesized that biorelevant *in vitro* drug dissolution testing combined with mechanistic kinetic modeling can be a tool to characterize the variability of human gastric motility in the context of oral capsule pharmacokinetics. A starting point for our analysis was pharmacokinetic data from a bioequivalence study with ritlecitinib (later referred to as API)—a novel JAK3/TEC inhibitor that exhibits rapid dissolution and rapid permeation.^13^ The present study’s goal was to propose experimental protocols that would explain the considerable variability of the time required to reach a maximal drug concentration in plasma (*T*_max_), which ranged from 0.5 to 2 h after capsule administration. To mimic the gastric motility *in vitro* regarding fluid flow rate and mechanical agitation, we used the recently developed apparatus, the *PhysioCell*.^14^ It combines the features of a flow-through cell with mechanical agitation. Through preprogrammed contractions, an elastic sleeve exerts stress on the dosage form located inside it, triggering dosage form disintegration. We wanted to investigate if such motility-mimicking protocols combined with a semimechanistic pharmacokinetic model focused on gastrointestinal dissolution and transit may help find the gastrointestinal motility-related factors influencing the *in vivo* dosage form performance.

## Materials and Methods

### Formulations

Ritlecitinib is a JAK3/TEC kinase inhibitor for the treatment of moderate to severe alopecia areata in individuals 12 years of age and older. The tosylate salt used for this study is highly soluble across the physiological pH range and the intrinsic solubility of ritlecitinib is 6.7 mg/mL. As the published experimental dissolution data show, ritlecitinib dissolution from a 100 mg capsule reaches approximately 100% within 30 min in all compendial dissolution media covering the pH range of 1.2–6.8.^15^ The p*K*_a_ of the molecule is 4.85, and log *P* is 1.55.^15^ Ritlecitinib is rapidly absorbed following oral absorption with an estimated oral bioavailability and fraction absorbed of 64 and 89%, respectively.^13^ The study included two immediate-release (IR) formulations (Pfizer, USA): tablet containing 50 mg and hydroxypropyl methylcellulose capsule containing 100 mg of ritlecitinib (API), microcrystalline cellulose, sodium carboxymethyl cellulose, and lactose. Both of the dosage forms contain no pH modifiers.

### The Clinical Trial

The clinical trial was a crossover study that included 123 healthy volunteers in total, who were administered 100 mg of API either as IR tablets (2 × 50 mg) or a capsule (1 × 100 mg) under fasting conditions. Pharmacokinetic data were obtained for 122 subjects after the tablet and 120 after capsule administration, respectively. 118 subjects had complete pharmacokinetic profiles for both the tablet and capsule. The blood sampling for PK took place 0.5, 1, 1.5, 2, 3, 4, 6, 12, 16, and 24 h after drug administration. All of the volunteers gave informed consent to participate in the clinical trial. The study protocol followed the principles of the Helsinki Declaration and was reviewed and approved by Advarra Independent Ethics Committee (reference number: 00000971). Volunteer demographics are presented in the Electronic Supporting Information (Table S1).

### Pharmacokinetic Characterization

**Figure 1:**
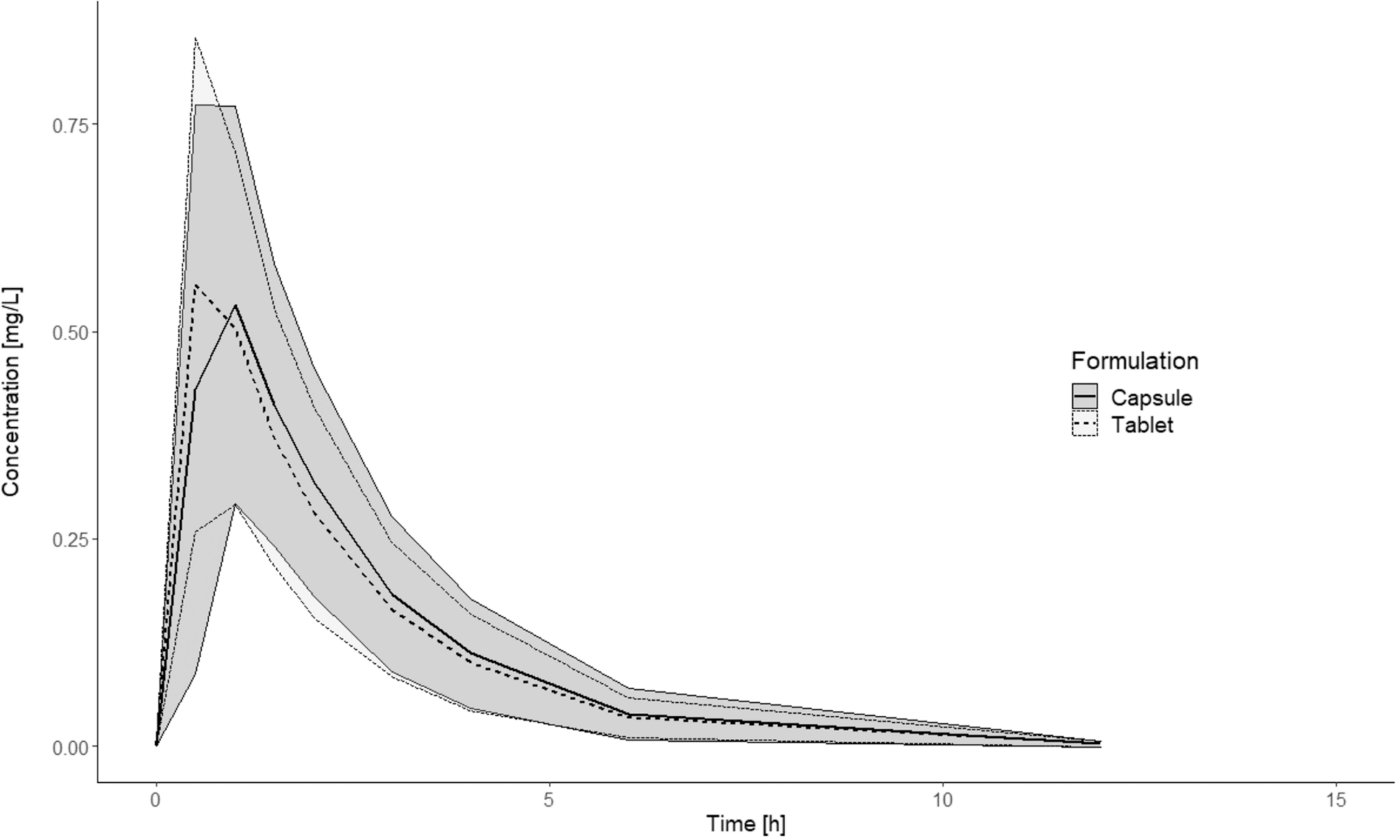
PK profiles obtained in the clinical trial after administration of the tablet (*n* = 122) and capsule (*n* = 120). Data are presented as means (lines) with one standard deviation (ribbons).

A noncompartmental analysis of the drug concentrations in plasma from the clinical trial, performed in R (v. 4.1.3) with *PKNCA* library (v. 0.9.5), showed that both *C*_max_ and AUC were bioequivalent between capsule and tablet formulations, but a difference in the *T*_max_ was noted (Electronic Supporting Information, Table S2). The capsule *C*_max_ occurred later than for the tablet—on average 1 h vs 0.5 h, respectively (Figure 1). Such a difference in the *in vivoT*_max_ was difficult to explain by simple dissolution experiments in a compendial apparatus because both formulations immediately released the API. To clarify this matter, we employed a novel approach to IVIVP by dividing the clinical study population into subpopulations of different gastric motility types, translating their characteristics into the language of physiologically driven dissolution test protocols and performing PK simulation to match the observed data.

### Population Pharmacokinetic Modeling

To better understand the disposition and elimination of the API, a fit-for-purpose population PK model was developed based on the plasma concentrations determined after tablet administration. It was justified by a very fast (ca. 1 min) and mechanical stress-insensitive tablet disintegration as well as the rapid dissolution and permeation of the API itself. The modeling was performed in the R software, with the *nlmixr* library (v. 2.0.6) and all dependencies. The model described how API concentrations changed over time after a single administration in healthy volunteers and allowed a reliable reflection of PK parameter combinations in the studied population.

Log-normal distribution of the pharmacokinetic parameters was assumed during the population PK model building. The modeling process followed the guidelines acknowledged by both industry and academia.^16,17^ Briefly, it was a step-by-step procedure, in which various structural and stochastic approaches were tested. At each step, a thorough visual examination of the standard goodness-of-fit plots was performed,^18^ as well as comparing the test functions, such as the decrease of log-likelihood or Akaike Information Criterion. Finally, the Visual Predictive Check (VPC) served as an internal validation tool.^19^

The following equation presents how interindividual variability (IIV) and covariate elements were expressed in the model:
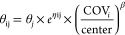
where θ_*ij*_ is a value of *j*-th pharmacokinetic parameter for *i*-th individual, θ_*j*_ is the population parameter estimate, η_*ij*_ is a random variable characterizing interindividual variability, COV_*i*_/center is the centered individual covariate value, and β is a scaling exponent.

It was found that a two-compartment model with a lag time, first-order absorption, and linear elimination best described the data set. A combined model with additive and proportional elements best described the residual unexplained error. Also, the body weight (centered at 70 kg) was a significant covariate for the apparent clearance from the central compartment (CL/*F*) and the volume of distribution in the central compartment (*V*_1_/*F*).

The final tablet population PK model was used to characterize the capsules. As the same subjects were included in the study, it was assumed that the parameters describing the disposition and their covariate dependencies were formulation-independent. The absorption type and its parameters were characterized separately. Several approaches were tested, including models with and without lag time (*t*_lag_), and with Erlang-type absorption. It was found that the model with first-order absorption rate constant (*k*_a_) and *t*_lag_ best describes the absorption from the capsules. The typical population parameter estimates (Table 1), along with their variabilities and correlations, were subsequently utilized for classifying individuals into gastric emptying types and in the IVIVP model. Additionally, the model included covariance between several interindividual variability elements (Electronic Supporting Information, Table S3).

**Table 1:** Final Population PK Model^a^

parameter		estimate	RSE [%]	BSV [%]	shrinkage [%]
tablet	*t*_lag_ [h]	0.157	6.00	77.86	21.26
	*k*_a_ [1/h]	5.08	6.96	107.0	16.05
capsule	*t*_lag_ [h]	0.36	3.60	28.88	16.89
	*k*_a_ [1/h]	4.88	6.58	117.34	13.62
CL/F [L/h/70 kg]		75.23	0.85	37.61	0.91
β_CL/F_, weight		1.21	18.8	-	-
V_1_/F [L/70 kg]		86.69	1.13	40.69	8.81
β_V1/F_, weight		0.73	28.2	-	-
Q/F [L/h/70 kg]		41.43	2.61	-	-
V_2_/F [L/70 kg]		31.38	1.91	25.75	36.31
additive error		0.70	-	-	-
proportional error		0.12	-	-	-

a β_CL/F_, β_V_1_/F_—scaling exponents for the apparent systemic clearance and apparent volume of the central compartment, respectively; BSV—between-subject variability; CL/*F*—apparent systemic clearance; *k*_a_—absorption rate constant; *Q*/*F*—apparent intercompartmental clearance; RSE—relative standard error; *t*_lag_—lag time; *V*_1_/*F*—apparent volume of the central compartment; *V*_2_/*F*—apparent volume of the peripheral compartment.

### Individual PK Profiles

Apart from the population PK modeling, a classical two-step method was used to obtain the individual predicted profiles of API in plasma after capsule administration. This approach provided greater flexibility in the model fitting, as optimizing the drug PK parameters in a particular healthy volunteer was independent of the other subjects. A two-compartment model with linear elimination was fitted to the observed PK profiles with a 1/*Y*_pred_^2^ weighting, using the *curve_fit* and *odeint* functions from the *scipy* library (v. 1.5.2) in Python (v. 3.8.5). The initial estimates of all of the PK parameters except the *t*_lag_ were the individual estimates obtained for the tablets in the posthoc population PK analysis. The *t*_lag_ initial estimate was the midpoint between (*t*_first_ - 0.5 h) and *t*_first_, where *t*_first_ denotes the time of the first measured concentration. To reflect the independence of the drug disposition of the oral dosage form type, the bounds of the clearances and volumes of distributions for the capsule PK fitting were set as ±20% relative to the tablet estimates.

### Subject Classification

The individual parameters were used to generate dense time–plasma concentration profiles that allowed a better understanding of the API’s behavior within the first two hours after administration of the capsule. For each subject, the occurrence of the experimental and model-predicted *C*_max_ was compared. Then, the subjects were binned into several categories that differed in terms of the absorption phase. We hypothesized that these groups could reflect how distinct gastric motility patterns influence the release of the API from the capsule, dissolution, and absorption.

Two pharmacokinetic experts independently reviewed the generated profiles and compared their observations. The final classification and the prevalence of each *in vivo* dissolution/absorption type in the population were used in further IVIVP simulations and comparisons with the clinical data.

### Biopredictive Dissolution

#### Apparatus

The biopredictive dissolution tests were performed in the *PhysioCell* apparatus. This novel device can simulate physiological mechanical agitation by applying a pressure wave on a dosage form through an elastic sleeve in the main dissolution compartment, the *StressCell*. Also, it can mimic variable kinetics of gastric emptying of noncaloric liquids by adjusting the medium flow rate by a peristaltic pump. Moreover, *PhysioCell* simulates the temperature gradient of the dissolution medium, as it has been proven to have an impact on capsules disintegration after fasted intake with a glass of water at ambient temperature.^20^ A detailed description of the apparatus’s operation principles and its functionalities is given elsewhere.^14^ In this study, *PhysioCell* was used in a closed-loop configuration, meaning that the dissolution medium was pumped from a heated and well-stirred medium reservoir into *StressCell* and circulated in the system. The sampling took place from the reservoir. The dissolution samples were automatically withdrawn through 1 μm polyethylene cannula filters and transferred for the measurement to the spectrophotometer every 2 min until the end of the test. The dissolution tests were performed in triplicates.

#### Biopredictive Dissolution Scenarios

To adequately reflect the intragastric conditions that triggered the API release from the capsule, the proposed dissolution protocols differed in terms of the scheduled behavior of *StressCell*, which simulated an *in vivo* gastric emptying event and intragastric mechanical stresses. The gastric emptying sequence consisted of three cycles: two pressure waves of 300 mbar and emptying of *StressCell* to the medium reservoir with the second wave, followed by rapid filling of *StressCell* with the medium (110 mL/min for 18 s).

All of the protocols utilized a dynamic change of the medium flow rate, starting with filling *StressCell* with the medium at the rate of 50 mL/min and then gradually decreasing to 8 mL/min at 14 min. Additionally, the medium temperature in *StressCell* increased gradually from an ambient value to 37 °C within the first 20 min of the experiment.

#### Media and Chemicals

The modified simulated gastric fluid (mSGF) served as a dissolution medium, reflecting the gastric environment after fasting intake of the formulation with a glass of mineral water. The mSGF consisted of mineral water *Żywiec Zdrój* (Żywiec Zdrój S.A., Warsaw, Poland), acidified to pH 2.0 with 25% HCl (VWR Chemicals, Leuven, Belgium). As provided by the manufacturer, the composition of the *Żywiec Zdrój* is hydrogen carbonates 121.06 mg/L, fluorides 0.07 mg/L, magnesium 5.37 mg/L, calcium 36.39 mg/L, and sodium 7.79 mg/L. Before the dissolution test, the dissolution medium was degassed by sonication for 15 min. As the API is highly soluble across a wide pH range and the solubility equals 27 mg/mL at pH = 2, the medium volume circulating in *PhysioCell* (500 mL) and even the fluid volume contained in *StressCell* only (34 mL) were sufficient to ensure sink conditions.

#### Analytical Method

The dissolution samples were analyzed using an Agilent 8453 UV–vis Spectroscopy System in closed-loop mode. The absorbance was measured using quartz flow-through cells with a 1 mm lightpath at 278 nm. The analytical method was found to be linear in the API concentration range of 0.04–0.20 mg/mL.

### IVIVP Modeling and Simulations

#### Software

The models for the *in vitro* drug dissolution and IVIVP were custom-built in Python (v. 3.8.5) with *numpy* (v. 1.19.2) and *scipy* (v. 1.5.2) libraries and their dependencies. Data were visualized in R with *ggplot* (v. 3.3.6) or in Python with *seaborn* (v. 0.11.2) or *matplotlib* (v. 3.4.3).

#### Dissolution Modeling

**Figure 2:**
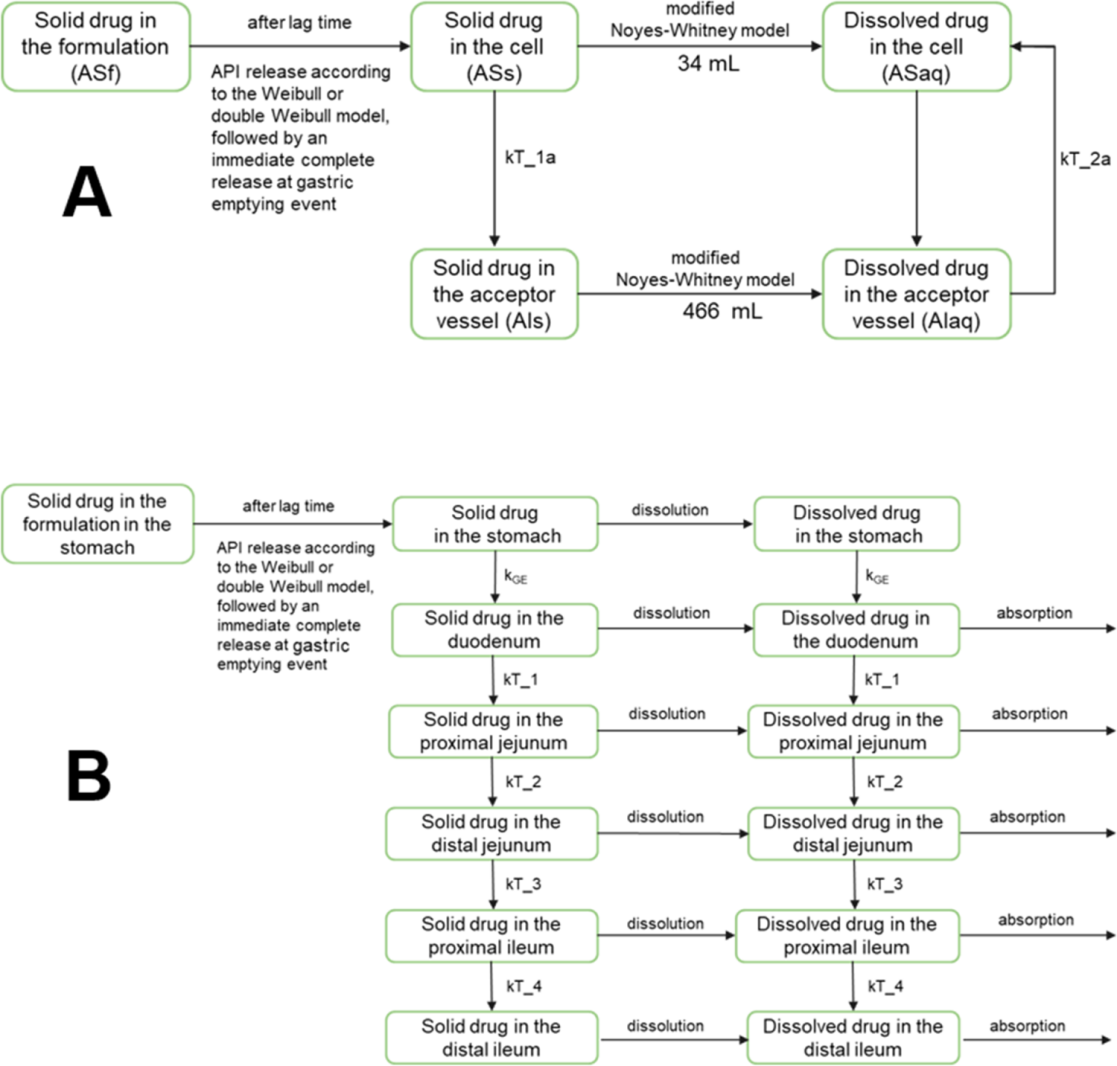
Models used in the IVIVP analysis. (A) Compartmental kinetic model of API dissolution in *PhysioCell*. The *kT*_1a and *kT*_2a represent the rate constants. (B) *In vivo* absorption-transit submodel. After absorption, the dissolved drug undergoes a two-compartment disposition, as defined in the population PK model. *k*_GE_ = gastric emptying rate constant; *kT*_1, *kT*_2, *kT*_3, and *kT*_4 = intestinal transfer rate constants.

The *PhysioCell* apparatus mimics physiological gastric conditions, including the mechanical stress exerted on a solid dosage form and mass transfer from the stomach to the intestine. Figure 2A presents a schematic representation of the dissolution model that accounted for the mass transfer in *PhysioCell*. The applied dissolution model accounted for the experimental flows in the compartments of the apparatus. Therefore, it was possible to characterize the dissolution process irrespective of the flow. The first-order mass transfer between *StressCell* (containing 34 mL of fluid) and the reservoir/acceptor vessel (containing 466 mL of fluid) was described with the rate constants *kT*_1a and *kT*_2a, of which values were derived from the instantaneous rate of the fluid flow in *PhysioCell*. The apparatus was used as a closed system. Therefore, the dissolved drug circulated between *StressCell* and the reservoir/acceptor vessel.

The relatively slow initial phase of the solid API release from the capsule, either spontaneous or induced by the intragastric stress, was described with the classical Weibull or double-Weibull model with a *t*_lag_. The choice of a double-Weibull over a classical Weibull model was based on the visual examination of the dissolution curve shape and comparison of the predicted vs observed dissolved drug amounts. In general, the curves with distinct inflection points were fitted with a double-Weibull model. For that purpose, the model was fitted to the mean *in vitro* dissolution profile obtained in each program, from the *t*_lag_ up to the last time before the simulated gastric emptying event.

The API dissolution from the tablet or capsule was parametrized with the modified Noyes–Whitney equation, according to Takano et al.:^21^
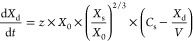
where d*X*_d_/d*t* is the dissolution rate; *z* is the dissolution coefficient; *X*_0_ is the initial amount of undissolved API, equivalent to the strength of the solid dosage form (100 mg); *X*_s_ is the amount of undissolved API at time *t*; *C*_s_ is the solubility of API (27 mg/mL); *X*_d_ is the amount of dissolved API at time *t*; and *V* is the volume of fluid (medium).

To find the optimal *z* value for the tablets, the modified Noyes–Whitney model was fitted to the mean observed amount of the dissolved API starting from *t*_lag_ to the beginning of the plateau phase. For the capsules, the *z* value was estimated by fitting the model to the three points included in the most pronounced GET-related API release of all dissolution results (i.e., Group 1).

#### IVIVP Assumptions and Setup

The IVIVP model included a mechanistic absorption-transit submodel (Figure 2B) and a drug disposition submodel. It followed the assumptions listed below:

- the gastrointestinal tract comprised six parts, including the stomach and five segments of the small intestine: duodenum, proximal jejunum, distal jejunum, proximal ileum, and distal ileum;
- the release of the solid drug from the capsule and dissolution of API in the gastric and intestinal media were parametrized with a combination of the (double) Weibull model and modified Noyes–Whitney model, analogously to the API dissolution in *PhysioCell*;
- the first-order gastric emptying rate constant (*k*_GE_) was variable and ranged from 1 to 14 1/h;^22−25^
- gastric emptying time (GET), that is the “housekeeper wave” time, was defined according to the dissolution scenario in *PhysioCell*; after this time, the *k*_GE_ was set at 100 1/h;
- dynamic fluid volume model—stomach and small intestine segments had a time-dependent fluid volume corresponding to the ingestion of 240 mL of water. The gastric fluid volume changed according to the *k*_GE_. The intestinal fluid volumes were described with polynomial equations that were fitted to the experimental data from Mudie et al.^22^ (Figure S1);
- mass transfer through the stomach and small intestine segments followed the first-order kinetics described with fixed rate constants;
- ritlecitinib did not precipitate from the solution and the solid of ritlecitinib tosylate did not change its form, for instance, by disproportionation to the free base or conversion into a hydrochloride or other salt;
- drug absorption occurred in the small intestine only. The effective permeability (*P*_eff_) was adjusted to 1.5 × 10^–4^ cm/s to match with the average clinical *T*_max_ of the tablets (0.5 h) at the average *k*_GE_ of 8.5 1/h;
- interindividual variability of the absorption rate constant (*k*_a_) from the population PK model was assigned to the duodenal fluid volume to reflect absorption variability;
- PK simulations included a varied number of subjects (12–100), interindividual variability of PK parameters as estimated in the population PK model, and no interoccasion variability.

The fitted parameters of the *in vitro* dissolution model served as direct input to the IVIVP model. Initially, the model outputs for the tested dissolution scenarios were compared for a small (*n* = 12) group of virtual individuals. The pharmacokinetic parameters of these individuals were randomly drawn from the population PK distribution. Also, the sensitivity of the model for various *k*_GE_ values (3–14 1/h) was determined. The small group was also used in the initial comparisons to the clinical results.

In the final simulations, a larger number of unique subjects (*n* = 100) was simulated for each drug dissolution scenario and each *k*_GE_ ranging from 1 to 14 1/h. Then, to account for the probability of each gastric motility scenario in the general population, we applied a randomization algorithm. First, the number of subjects from each group had to reflect the prevalence of a given gastric motility type. Then, each subject was randomly assigned to the scenario defined with the dissolution group and *k*_GE_. The procedure was repeated 10 times, and the mean time–plasma concentration curves and variabilities were compared with the clinical trial results.

## Results

### Capsule PK Profiles Translated to *In Vitro* Dissolution Tests

**Figure 3:**
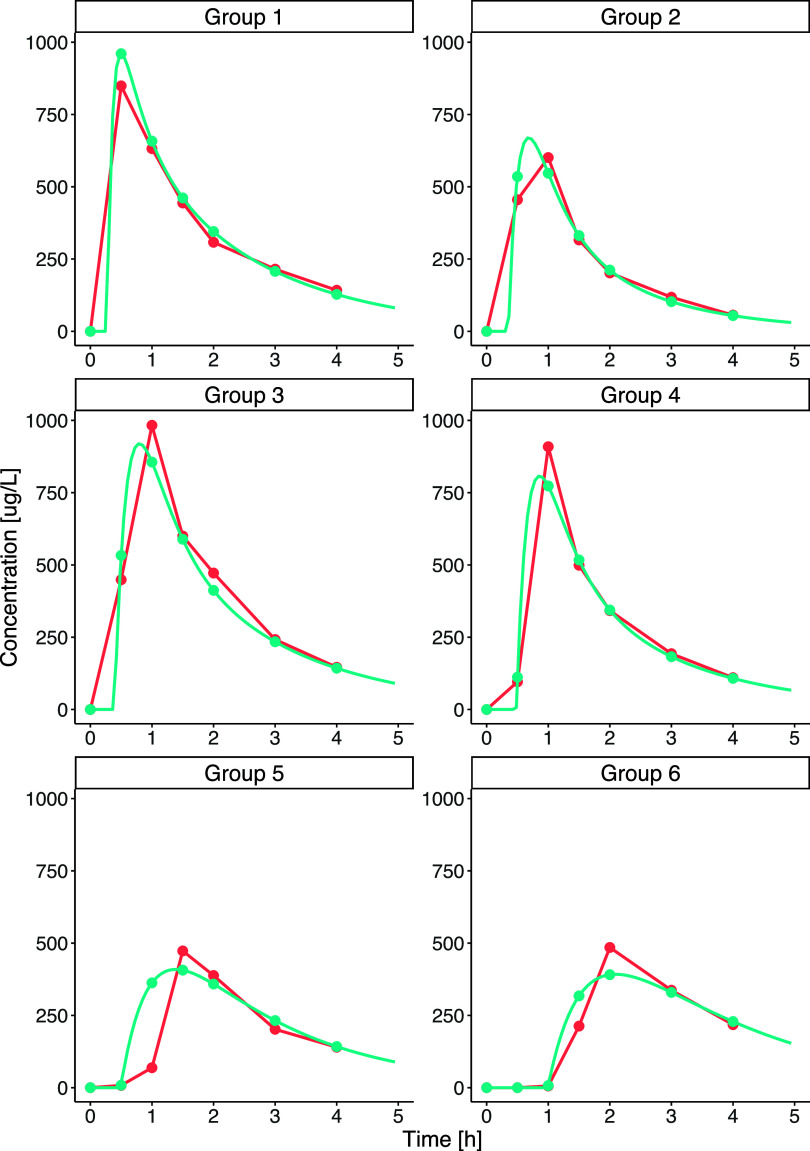
Examples of the PK profiles representing the six gastric motility categories (Groups 1–6). Red lines present the concentrations determined *in vivo*, while blue lines present the concentrations simulated from the individual parameters calculated from the PK model.

**Figure 4:**
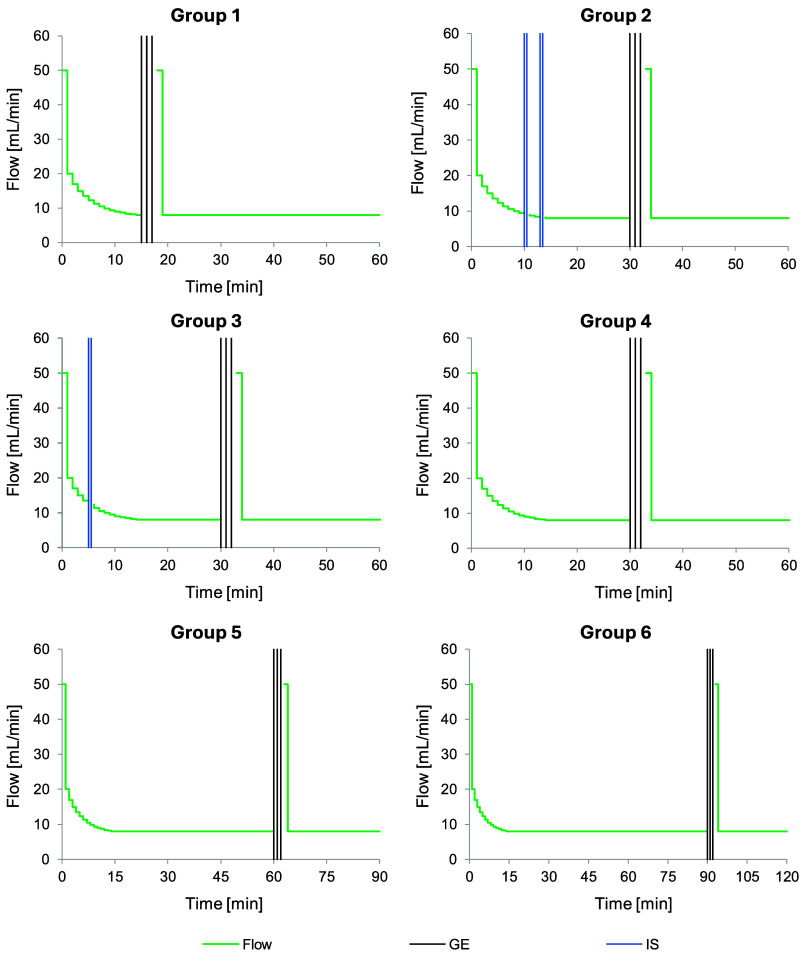
*PhysioCell* dissolution programs designed for the capsules to reflect the possible physiological gastric emptying kinetic patterns. The green lines represent the medium flow rate in mL/min; the vertical blue lines represent the intragastric stresses, and the vertical black lines represent the complete gastric emptying events with the mass transfer from *StressCell*.

The PK analysis of the API plasma concentrations after capsule administration provided 118 rich-point individual PK profiles. Owing to the rapid dissolution and permeation properties of the API, analysis of the absorption phase of the PK profiles revealed the most probable capsule behavior in the stomach: the spontaneous disintegration of the capsule shell or disintegration in response to mild or moderate stomach muscle contractions in interdigestive migrating motor complex (IMMC) phase II and “housekeeper wave” in IMMC phase III.^10^ Based on the *T*_max_, we identified the onset of the IMMC phase III, i.e., complete gastric emptying time (GET), while from the magnitude of the drug concentration at the first sampling point of 0.5 h, we proposed the time and intensity of intragastric stresses in IMMC phase II. We recognized six distinct gastric motility patterns (Table 2). Figure 3 presents examples of the individual PK profiles representing each gastric motility subcategory. These categories served as a basis for the custom-design of six *in vitro* dissolution programs in *PhysioCell* (Figure 4).

**Table 2:** Characteristics of the Capsule PK Profiles and Corresponding *In Vitro* Dissolution Tests in *PhysioCell*^a^

	*in vivo* PK profile		*in vitro* dissolution test		
group	*T*_max_ [h]	*C*_0.5 h_ [mg/L]	intragastric stress	GET [min]	prevalence (%)
1	≤0.5	not applicable	none	15	27
2	0.5–1.0	0.6–0.95 *C*_max_	300 mbar at 10 and 13 min	30	22
3	0.5–1.0	0.3–0.6 *C*_max_	200 mbar at 5 min	30	18
4	0.5–1.0	<0.3 *C*_max_	none	30	13
5	1.0–1.5	not applicable	none	60	13
6	>1.5	not applicable	none	90	7

a *C*_0.5 h_—drug concentration in plasma at 0.5 h; GET—complete gastric emptying time (“housekeeper wave” time).

### Biopredictive Dissolution Curves

**Figure 5:**
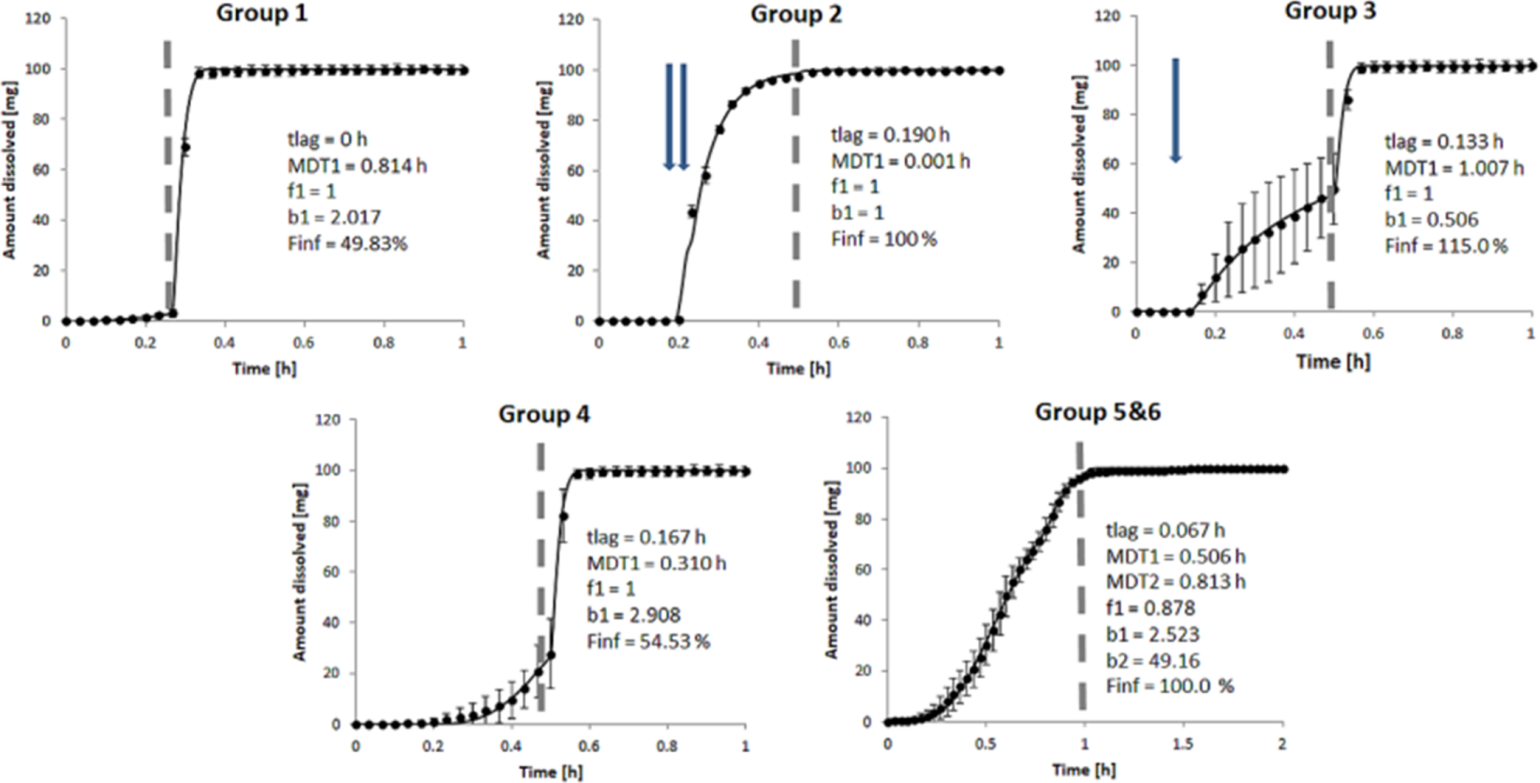
Goodness-of-fit plots for the mechanistic model of drug dissolution from capsules in *PhysioCell*. Experimental data are presented as means (dots) with standard deviation (bars), while the model predictions are shown as lines. The dashed gray line symbolizes the complete gastric emptying event, and the arrows represent the intragastric stresses. The experiments were replicated three times, except for Group 5&6 with *n* = 6, due to pooling the data from Groups 5 (GET 60 min) and Group 6 (GET 90 min).

The novel apparatus for testing drug dissolution *in vitro*—*PhysioCell*—is capable of simulating pressure waves, fluid flow rates, temperature, and pH gradients, similar to those found in the gastrointestinal tract.^14^ Therefore, we chose *PhysioCell* as an optimal tool for evaluating capsules’ behavior under the six designed test protocols differing in time and intensity of simulated intragastric pressure waves and GET. Figure 5 presents the biopredictive dissolution curves for the capsules in the Group 1–6 tests listed in Table 2.

The Group 1, 4, 5, and 6 scenarios included no intragastric stress events but only a complete gastric emptying event in the form of three 300 mbar pressure waves combined with forced mass efflux, simulated at 15, 30, 60, and 90 min, respectively. Under these conditions, the capsules started disintegrating spontaneously within approximately 10 min. After that, in Groups 1 and 4, the relatively early gastric emptying caused a burst release of the remaining solid API from the capsule and complete dissolution. Under the scenarios with the late gastric emptying waves (Groups 5 and 6), the capsule spontaneously opened and completely dissolved API in the same pattern, already before the emptying event (Figure S2 in the Electronic Supporting Information). Therefore, we pooled the profiles from these two scenarios into the new Group 5&6 (*n* = 6). Moreover, the two scenarios with the simulated intragastric stress events (Groups 2 and 3) demonstrated a distinct susceptibility of the capsules to the early pressure waves of different intensities. Two pressure waves of 300 mbar applied at 10 and 13 min (Group 2) caused the capsules to release the API completely. On the other hand, the single pressure wave of lower intensity (200 mbar), applied at 5 min in the Group 3 scenario, initiated the capsule disintegration, but complete API release and dissolution occurred only after the major gastric emptying event. This scenario was also associated with a highly variable drug dissolution rate before the GET.

### Parameterization of a Mechanistic *In Vitro* Drug Dissolution Model

To parametrize the performance of API in *PhysioCell*, we used a mechanistic kinetic model that involved the release of the solid drug from the capsule (according to Weibull or double-Weibull model), dissolution of the solid particles in the medium (modified Noyes–Whitney model with the *z*-factor), and transfer of the solid and dissolved substance between the compartments of the apparatus. The submodels that we chose for the capsule disintegration and drug dissolution are common approaches in PBBM.^7−9,26,27^Figure 5 presents the full model fitting to the dissolved drug amounts determined in the medium circulating in the apparatus. The model described well the behavior of the capsules in *PhysioCell* in all of the *in vitro* dissolution scenarios. Therefore, the optimized parameters for the Noyes–Whitney model (dissolution coefficient *z* = 1.08 mL/mg/h) and Weibull or double-Weibull models could be further used in the IVIVP model to simulate the drug concentrations in the healthy volunteers’ plasma after the capsule administration.

### IVIVP results

#### Influence of Intragastric Stresses and Gastric Emptying Kinetics on Simulated PK Profiles

The mechanistic IVIVP model involved all of the processes that an API administered in the form of an oral capsule can experience in the body: formulation disintegration and drug dissolution in the stomach and several compartments of the small intestine, mass transfer through the GIT, and drug absorption from the small intestine to blood followed by drug distribution and elimination. The drug disposition and its intersubject variability were derived from the population PK model built for the tablets at the earlier stage of the work. Such coupling of mechanistic drug dissolution and absorption description with population PK has been recently proven as an efficient IVIVP approach.^26,27^ Population PK enables a comprehensive description of the disposition phase. It defines not only the typical PK parameter values in the studied population but also their interindividual and interoccasion variabilities. Identifying the significant covariates and correlations between the elements describing the variabilities allows more reliable predictions of drug exposure.^26,27^

The developed IVIVP model could differentiate between the proposed dissolution programs in the initial test group, comprising 12 virtual subjects. It allowed the visualization of the possible variability in drug pharmacokinetics within a small study group. We intentionally removed the residual error effect from the model’s population PK part to clarify the simulated plasma profiles better. Each dissolution program led to distinct outcomes, and the resultant profiles differed in the timing and magnitude of model-estimated drug exposure (Figure S3, Electronic Supporting Information). It is worth noting that in the PK simulations, Groups 5 and 6 were presented separately because their different GET (60 and 90 min, respectively) gave different results with the physiological rates of stomach-to-intestine mass transfer, which are lower than the ones in *PhysioCell* (on average 3–14 1/h vs 14–88 1/h depending on the programmed flow rate).^14,22−25^

**Figure 6:**
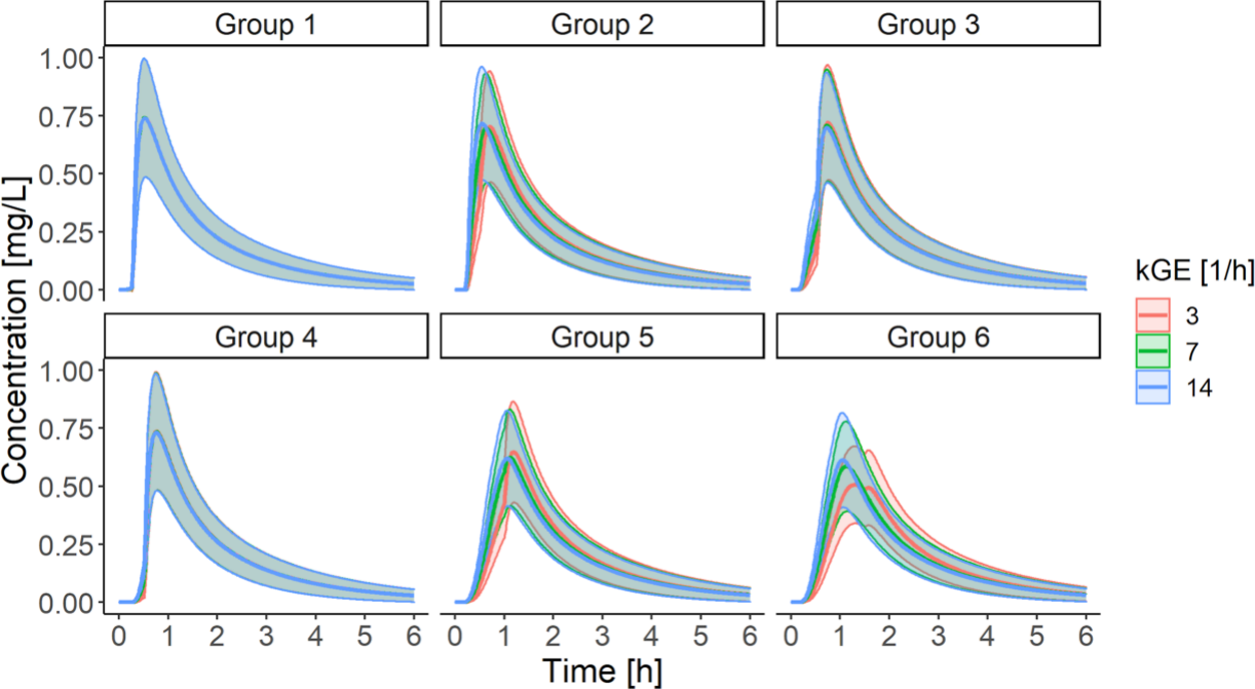
Comparative analysis of the influence of *k*_GE_ on the simulated drug concentration–time profiles according to the selected dissolution experimental setup. Data are presented as means with standard deviation as ribbons (*n* = 12).

In the next stage, we tested the sensitivity of the outcomes to the gastric emptying kinetics; we compared how the mean drug dissolution profiles obtained from the Group 1–6 tests would translate to the drug concentration in plasma after varying the continuous fluid flow-related first-order gastric emptying rate constant (*k*_GE_). Figure 6 presents the results for the three *k*_GE_ levels found in the literature: 3 (low), 7 (medium), and 14 (high) 1/h.^22−25,28^

The analysis indicated that *k*_GE_ was an influential parameter in some drug dissolution scenarios, representing specific gastric motility patterns. Groups 1, 3, and 4 produced more or less consistent PK profiles regardless of the *k*_GE_ value. In Groups 2, 5, and 6, different *k*_GE_ values gave divergent outputs, especially within the first hour; these differences were even more pronounced in real-sampling PK profiles that presented only the drug concentrations at the times of blood collection in the clinical trial (results not shown). It suggested that the gastric fluid outflow was a factor limiting the rate of drug absorption, at least before the GET. Of note, the concentrations simulated for Groups 5 and 6 were identical at the medium and high *k*_GE_. Most probably, the majority of the API had already evacuated from the stomach by the time of the gastric emptying, assumed at 60 or 90 min for Group 5 and Group 6, respectively. Further exploration of these groups showed that a late major gastric emptying event caused a double drug concentration peak phenomenon when the *k*_GE_ was the slowest (Figures 6 and S4 in Electronic Supporting Information).

The simulations showed that tablet’s dissolution did not depend on the stress patterns, and all of the dissolution scenarios led to comparable PK profiles (Electronic Supporting Information, Figure S5). Hence, further analysis focused on the capsule only.

#### The Final IVIVP Model Estimations and Comparison with the Clinical Data

The final simulations and comparison with the clinical data demanded a larger group of subjects (*n* = 100) to account for intersubject variability and the prevalence of specific gastric kinetic patterns in the population. Although the simulations and observations aligned well for Groups 1, 3, and 4, there was a misspecification for Groups 2, 5, and 6. Briefly, for Group 2, the first sampling point at 0.5 h was overestimated, while in Group 6, the simulated *C*_max_ was too early compared with the clinical results. Also, for both Groups 5 and 6, the model underestimated the drug concentration in plasma at the first measured point (0.5 h).

We hypothesized that capturing a relatively low concentration at 0.5 h for Group 2 should require constraining the *k*_GE_ to the lowest value of 3 1/h. This approach allowed for better representation of the clinical data.

A similar technique better approximated the late peak concentration in Group 6. Still, *k*_GE_ = 3 1/h was too high to capture the late *T*_max_ in Group 6 adequately. Therefore, we performed an additional investigation with *k*_GE_ = 2 1/h. Only after the slower emptying was imposed for Group 6 did the API concentrations increase steadily until GET, and then peaked after the emptying event. It suggested that the API can reside in the stomach for a prolonged period in individuals with poor gastric motility. To the best of our knowledge, the *k*_GE_ as low as 2 1/h was not recorded as a mean value in the available research data on the gastric emptying of a noncaloric liquid (the reported mean *k*_GE_ ranged from 2.8 1/h to 14 1/h). However, considering the physiological variability, the lower *k*_GE_ values in individual subjects cannot be excluded and may occur in the population.^22−25,28^

**Figure 7:**
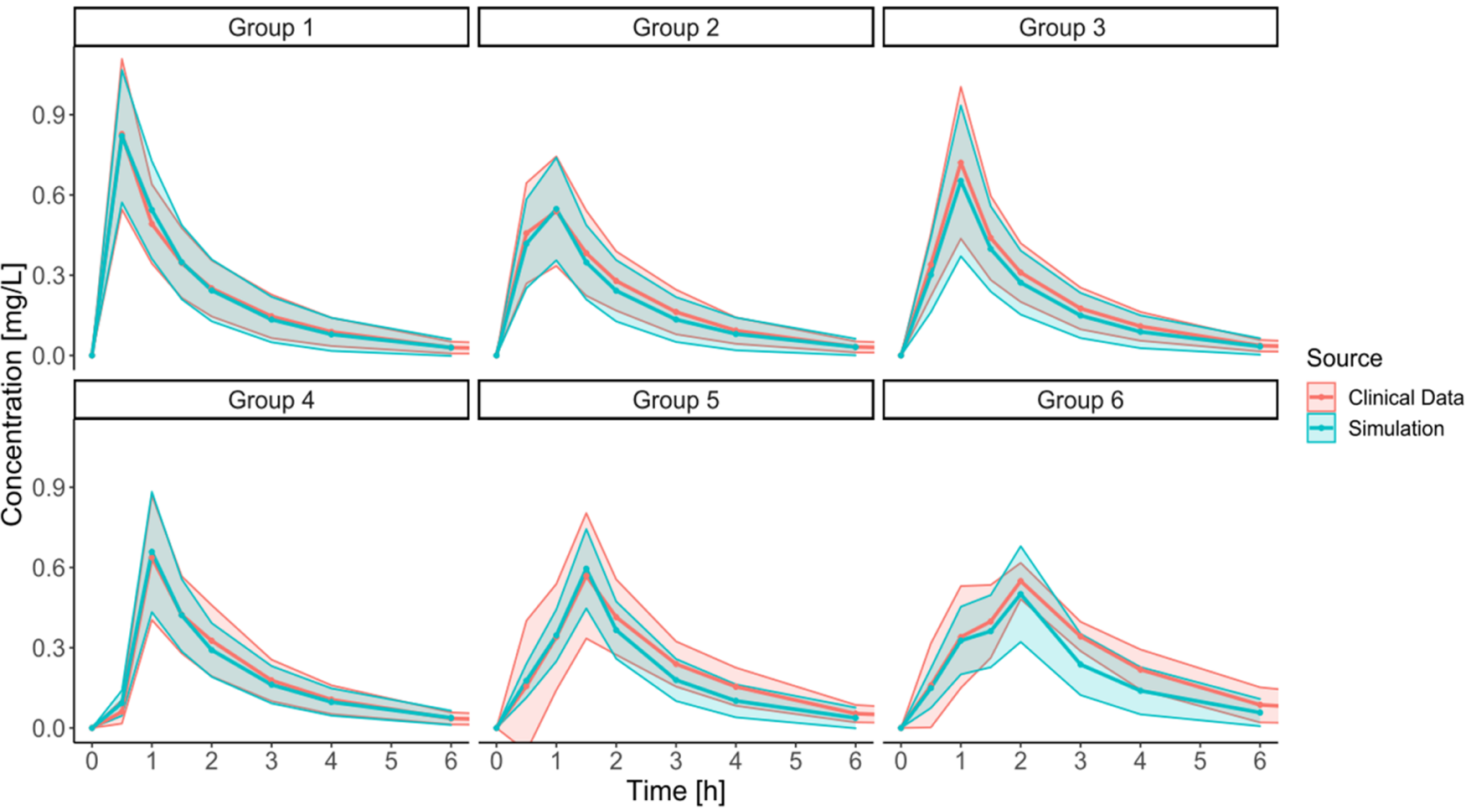
Final IVIVP model simulations (*n* = 100) comparison with the clinical trial outcome, stratified into specific gastric motility types. Data are presented as means (line) with standard deviation (SD) values (ribbons). The following *k*_GE_ distribution was used in the simulations: 3 1/h for Group 2, 2 1/h for Groups 5 and 6; otherwise, a random *k*_GE_ value from the 3–14 1/h range. The final Groups 5 and 6 involved the intragastric stresses from Group 3.

Lastly, the higher drug concentrations in plasma at 0.5 h for Groups 5 and 6 recorded in the clinical trial indicated that despite the late occurrence of the IMMC phase III wave, early gentle intragastric stresses in IMMC phase II might trigger the formulation’s disintegration and dissolution. For this reason, the improved simulation outcomes for Groups 5 and 6 relied on the dissolution parameters obtained from Group 3, but with GET kept at 60 and 90 min, respectively. Only after introducing this assumption, the simulations reflected the observed data. It is worth emphasizing that such a hybrid scenario could not be performed experimentally in *PhysioCell* due to insufficient mass transfer from the *StressCell* occurring at the fluid flow rates below 8 mL/min (equivalent to the *k*_GE_ of 14 1/h) in the upward direction.^14^Figure 7 presents the final output of the developed IVIVP models: one of 10 draws from the virtual 100 individuals, randomized according to the prevalence of the gastric kinetics type and the *k*_GE_ value characteristic for the given subtype.

## Discussion

In this study, we confirmed the hypothesis that physiologically driven *in vitro* dissolution experiments coupled with PK modeling and simulations constitute a tool for understanding the relationship between the human gastric motility in a fasted state and performance of an IR solid dosage form. In particular, based on the observed PK profiles, we recognized six patterns of gastric motility and rationally translated them into a series of *in vitro* dissolution tests. A combination of several elements enabled the development of the novel methodology: rapidly dissolving and permeating API, mechanical stress-sensitive formulation (capsule), a large PK data set from the clinical trial comprising 118 PK profiles, use of innovative apparatus for physiologically relevant dissolution tests (*PhysioCell*), and IVIVP modeling and simulations.

The operating conditions of *PhysioCell* were designed to mimic the known factors acting on an oral solid dosage form *in vivo*, which was previously measured with telemetric capsules,^29−31^ magnetic resonance imaging,^22,32−34^ and gamma scintigraphy.^35^ In particular, the apparatus simulated the mechanical forces exerted upon a dosage form during gastric residence. These included mild or moderate IMMC phase II pressure waves—in the form of single or double stress events of 200 and 300 mbar, and IMMC phase III “housekeeper wave”—as triple 300 mbar stress combined with a forced mass transfer out of the “stomach” compartment.^36^ Moreover, the device mimicked the gradual flow rate of fluids emptied from the stomach.^23^ Of note, all parameters of the device were strictly controlled, including the fixed position of a dosage form, which is often an issue in other noncompendial devices.^37^ These physiologically driven, consistent, and nonrandom conditions of drug dissolution obtained with *PhysioCell* provided a foundation to understand how the variability of the gastric motility *in vivo* relates to the PK of the capsule in the clinical trial.^14,27^Table 3 summarizes the prevalence of the resultant gastric motility patterns in the population, their characteristics, and their impact on the drug concentration profiles in plasma. The interplay between the time of appearance and magnitude of intragastric stress events in the IMMC phase II, the time of the IMMC phase III (GET), and the *k*_GE_ value governs whether the capsule disintegration, gastric emptying, or API properties (dissolution or permeation) limit the rate of drug absorption. We found that in half of the population (Groups 3–5), the GET occurred late enough (0.5 h or later), and prior intragastric contractions were weak enough to make the capsule opening a rate-limiting step in the drug absorption. This information creates an appreciable space for researchers and formulation specialists. By tweaking the dosage form’s properties and susceptibility to the early-stage intragastric stresses, they could influence the product PK, e.g., *C*_max_ and *T*_max_.

**Table 3:** Overview of Proposed Gastric Motility Patterns and Their Influence on the Capsule Pharmacokinetics^a^

group (% in the population)	key features	approximate GET	PK consequences	explanation
1 (27%)	early GET (“sprint stomach”)	0.25 h	the highest *C*_max_ that occurs early after administration (about 0.5 h)	0/1 situation: capsule emptying occurs only at GET (immediately); neither capsule emptying nor gastric fluid flow is a limiting factor for drug absorption (API dissolution or permeation limits the absorption rate)
2 (22%)	normal GET, slow gastric fluid flow, strong intragastric stress(“tight stomach”)	0.5 h	concentrations at early sampling points are relatively high and the *C*_max_ occurs between 0.5 and 1 h	a strong intragastric stress occurs when the capsule is prone to mechanical agitation leading to complete and fast capsule opening; gastric fluid outflow is a limiting factor for drug absorption until GET
3 (18%)	normal GET, weak intragastric stress (“average stomach”)	0.5 h	concentrations at early sampling points are moderate and the *C*_max_ occurs between 0.5 and 1 h	A mild intragastric stress occurs when the capsule is prone to mechanical agitation; capsule emptying is relatively slow, thus being a limiting factor for drug absorption until GET
4 (13%)	normal GET, tiny intragastric stress (“gentle stomach”)	0.5 h	concentrations at early sampling points are very low and the *C*_max_ occurs between 0.5 and 1 h	the intragastric stress is negligible or occurs when the dosage form has already disintegrated; spontaneous capsule emptying is very slow (especially at the beginning), thus being a limiting factor for drug absorption until GET
5 (20%)	late GET, slow gastric fluid flow, tiny intragastric stress (“lazy stomach”)	1 h or later	the PK profile is flattened and/or the *C*_max_ occurs at 1.5 h or later	a mild intragastric stress occurs when the capsule is prone to mechanical agitation; capsule emptying is relatively slow, thus being a limiting factor for drug absorption until GET; long capsule emptying at an almost constant rate (zero-order-like) flattens the PK profile until GET, after which the drug concentration peaks

a GET—complete gastric emptying time (“housekeeper wave” time).

To the best of our knowledge, the comprehensive approach developed in this study has never been implemented before for the fasting intake of oral solid dosage forms. Currently, a few biorelevant dissolution apparatuses are available on the market or in academic use, offering the advantages of customized dissolution scenarios. One example of such a device is GastroDuo, an apparatus simulating gastric conditions *in vitro*. It was used to study the impact of gastric motility on IR dosage forms, but the investigators focused on the postprandial conditions only.^38,39^ Based on the clinical trial results, three types of gastric motility were proposed with regard to the so-called *Magenstrasse*, a phenomenon of fast gastric emptying of noncaloric liquids despite meal ingestion. Similarly to our study, Takagi et al. investigated the fasted state and utilized biorelevant dissolution.^40^ The authors used the Stomach-to-Intestine Fluid Changing (SIFC) system, an apparatus reproducing the pH shift that occurs *in vivo* during the transition from gastric to intestinal conditions, to explain the PK variability. This approach is much simpler than the one described in the present study. Three programs of the pH changes were applied to examine the premilled dosage form of a weakly basic drug, and no mechanical agitation was included in the dissolution tests. Also, this study did not link the prevalence of the proposed gastrointestinal pH changes in the SIFC model to the occurrence in the study population. Talattof et al. developed a Motility-Dependent Compartmental Absorption and Transit (MDCAT) mechanistic model to show by simulation how ingestion of 50 and 200 mL of solution translates into the variability in plasma profiles of BCS class I and III drugs with short and long elimination half-lives.^41^ This approach randomized the time of drug administration relative to that of the IMMC phase. Also, the rate and lag time of gastric emptying varied according to the IMMC phase timing. The model fitting parametrized the gastric motility characteristics to the older experimental data from seven subjects who received phenol red solution.^42^ Also, the simulations included a uniform distribution of the model variables (e.g., length of gastric cycle phases and phase I gastric emptying rate).^41^ Similarly to our observations that focused on a well-soluble and rapidly absorbed molecule, BCS class I APIs were sensitive to gastric emptying kinetics, especially if their elimination was rapid. The advantage of our approach is that we use the data from a larger cohort and assess the prevalence of each gastric motility type. Hens et al. studied *in vivo* dissolution of the BCS class II drug combined with monitoring of pH, fluid buffer capacity, and pressure in the GIT, following administration of ibuprofen oral IR tablets at fasting and fed conditions in 37 subjects.^43^ The authors found a strong negative correlation between the observed drug *C*_max_ and the time to IMMC phase III contractions postdose. Also, they concluded that future studies should focus on explaining how the other GIT features, including the amplitude of contractions and the gastric emptying rate, affect the variability of drug concentrations in plasma. Our study not only provides such data for an IR capsule (Tables 2 and 3) but also brings the methodology and workflow for investigating the other oral formulations.

Our study has several limitations. First, only one API and one pressure-sensitive formulation served as a base to design the *in vitro* dissolution tests in *PhysioCell* and characterize the gastric motility, including the time and magnitude of intragastric stress events, GET, and the gastric fluid outflow rate. Further studies with the other APIs or formulations are required to confirm the applicability of the dissolution scenarios (Table 2) and gastric motility group characteristics (Table 3). Second, gastric motility was not recorded directly in the clinical trial but rationally deduced from the PK profiles, assuming rapid and complete API absorption. Hence, all of the resultant gastric motility patterns approximate the given individual’s physiological state. It is, however, worth noting that among the identified gastric motility patterns, the 0.5 h GET occurred most frequently (53% of all cases; Table 3). It agrees with the direct measurements of the IMMC phase III in a fasted state.^29,44^

Although this study is limited to only one formulation, we believe this methodology can be implemented elsewhere and increase the biopredictive power of dissolution testing in the pharmaceutical industry. For example, the dissolution test scenarios can help predict the dosage form behavior *in vivo* before a phase 1 clinical trial. They may also help detect the subtle but potentially significant differences in response to gastric-related mechanical stress between the different batches of the drug product or between the test (generic) and reference (innovator) formulations. It should be pointed out that capturing the impact of stress events on the capsule disintegration cannot be efficiently detected in compendial apparatuses like USP2 or USP4. Such data may serve as an input for PBBM and patient-centric evaluation of medicinal products, for example, the refinement of the bioequivalence “safe space” for gastric stress-sensitive IR dosage forms.^7−9^ A particular field of application is bridging IR tablets and hard or soft capsules. Our recent studies showed that the latter are more prone to varied timing and magnitude of mechanical agitation in the stomach.^45,46^ The bridging may be challenging, especially for the products containing a rapidly absorbed and eliminated drug substance. Any triggering or slowing of the dissolution of the drug with such properties will be reflected in its plasma concentration profiles. Lastly, the prevalence of the gastric motility types (Table 3) adds another dimension to the variability that can be inputted in the IVIVP models and can help explain the performance of oral solid dosage form after fasted intake. One may recognize how the variability of gastric motility contributes to the variability of the PK profiles for a particular product containing the investigated drug substance, knowing that, for instance, the probability of very early gastric emptying is 27% (Group 1), with little relevance to the gastric fluid outflow to the duodenum. When the formulation is administered a second time, the same subject may be classified as a “lazy stomach” Group 5 representative (20% prevalence), with slow gastric emptying of the fluid and late GET. Using the proposed subgroups of gastric motility may help to answer the question on how key PK parameters may vary within the same individual solely due to the differences in gastric emptying kinetics.

## Conclusions

The study provides a unique combination of biopredictive dissolution testing with physiologically based modeling. We were able to derive subpopulations of specific gastric motility patterns from the clinical trial and recreate these patterns *in vitro*, utilizing a novel apparatus—*PhysioCell*. The simulations built upon the dissolution results and an advanced IVIVP model matched the clinical data, confirming the applicability of such an approach. In summary, it is an innovative integration of biopredictive dissolution methodologies with physiologically based PK modeling—the matter emphasized in the recent commentary of the European Union OrBiTo (Oral Biopharmaceutics Tools) project research group.^47^

## Data Availability

Upon request, and subject to review, Pfizer will provide the data that support the findings of this study. Subject to certain criteria, conditions, and exceptions, Pfizer may also provide access to the related individual deidentified participant data. See https://www.pfizer.com/science/clinical-trials/trial-data-and-results for more information.

## Supporting Information Available

Basic subject demographics in the clinical trial, main PK parameters calculated for the tablet and capsule administered in the clinical trial, correlation matrix of the interindividual variability elements included in the final population PK model, small intestinal fluid volumes from healthy adult volunteers fitted with the cubic equations in the initial phase and the average value in the later phase, dissolved amounts of API in the Group 5 and 6 dissolution tests performed for the capsules in *PhysioCell*, comparison of the simulated capsule PK profiles for all of the tested dissolution programs and fast gastric emptying (*k*_GE_ = 14 1/h), comparative analysis of the *k*_GE_ influence on the simulated individual PK profiles for 12 virtual subjects representing Group 6, and PK profiles simulated for 12 virtual subjects after the tablet administration (PDF)
