# Supplementary material for: More Than a Gut Feeling—A Combination of Physiologically Driven Dissolution and Pharmacokinetic Modeling as a Tool for Understanding Human Gastric Motility
Source: Mol Pharm. 2024 Jul 3;21(8):3824–37. doi: 10.1021/acs.molpharmaceut.4c00117 (PMC11345944; doi:10.1021/acs.molpharmaceut.4c00117)
Supplement: Supplementary file 1 — mp4c00117_si_001.pdf [file mp4c00117_si_001.pdf]

**Supporting Information**

# More than a gut feeling - a combination of physiologically-driven dissolution and pharmacokinetic modeling as a tool for understanding human gastric motility

Michał Romański [a,§,*], Marcela Staniszewska [b] Justyna Dobosz [b], Daria Myslitska [b], Jadwiga Paszkowska [b], Bartosz Kołodziej [b], Svitlana Romanova [b], Grzegorz Banach [b], Grzegorz Garbacz [b], Inese Sarcevica [c], Yeamin Huh [d], Vivek Purohit [d], Mark McAllister [c], Suet M. Wong [c], Dorota Danielak [a,§]

[a] *Department of Physical Pharmacy and Pharmacokinetics, Poznan University of Medical Sciences, 3 Rokietnicka St., 60-806, Poznań, Poland.*
[b] *Physiolution Polska, 74 Piłsudskiego St., 50-020 Wrocław, Poland.*
[c] *Worldwide Research and Development, Pfizer R&D UK Ltd., Sandwich, U.K.*
[d] *Worldwide Research and Development, Pfizer Inc., Groton CT, USA*

* Michał Romański, Department of Physical Pharmacy and Pharmacokinetics, Poznan University of Medical Sciences, 3 Rokietnicka St., 60-806, Poznań, Poland; michalroman@ump.edu.pl

§ M.R. and D.D. contributed equally to this paper.

Table S1. Basic subject demographics in the clinical trial. Data are presented as means with standard deviations

| Parameter | Value |
|---|---|
| Number of subjects | 123 |
| Age [years] | 33.1 ± 9.5 |
| Height [cm] | 173.7 ± 9.2 |
| Weight [kg] | 76.1 ± 12.3 |

Table S2. Main PK parameters calculated for the tablet and capsule administered in the clinical trial

| Parameter | Capsule | | Tablet | |
|---|---|---|---|---|
| | Mean ± SD | Median | Mean ± SD | Median |
| $AUC_{0-t}$ [μg×h×$L^{-1}$] | 1363.0 ± 540.8 | 1285.7 | 1311.40 ± 534.4 | 1211.5 |
| $AUC_{0-inf}$ [μg×h×$L^{-1}$] | 1371.38 ± 538.2 | 1283.1 | 1324.14 ± 529.3 | 1182.8 |
| $C_{max}$ [μg×$L^{-1}$] | 667.84 ± 264.2 | 612.0 | 660.04 ± 247.0 | 624.0 |
| $t_{max}$ [h] | 0.99 ± 0.41 | 1.00 | 0.74 ± 0.32 | 0.50 |

$AUC_{0-t}$ - area under the time-concentration curve measured to the last experimental data point, $AUC_{0-inf}$ - area under the time-concentration curve extrapolated to infinity, $C_{max}$ - maximum concentration, SD - standard deviation, $t_{max}$ - time to maximum concentration.

Table S3. Correlation matrix of the interindividual variability elements included in the final population PK model. The interindividual variability elements for the absorption phase ($t_{lag}$ and $k_a$) are presented separately for the tablet and capsule. The other parameters are shared between both formulations

| | $\eta_{tlag\_tablet}$/$\eta_{tlag\_capsule}$ | $\eta_{ka\_tablet}$/$\eta_{ka\_capsule}$ | $\eta_{CL/F}$ | $\eta_{V1/F}$ | $\eta_{V2/F}$ |
|---|---|---|---|---|---|
| $\eta_{tlag\_tablet}$/$\eta_{tlag\_capsule}$ | 0.688/0.283 | -0.589/-0.289 | - | - | - |
| $\eta_{ka\_tablet}$/$\eta_{ka\_capsule}$ | -0.589/-0.289 | 0.688/0.931 | - | - | - |
| $\eta_{CL/F}$ | - | - | 0.364 | 0.797 | 0.021 |
| $\eta_{V1/F}$ | - | - | 0.797 | 0.391 | -0.348 |
| $\eta_{V2/F}$ | - | - | 0.021 | -0.347 | 0.253 |

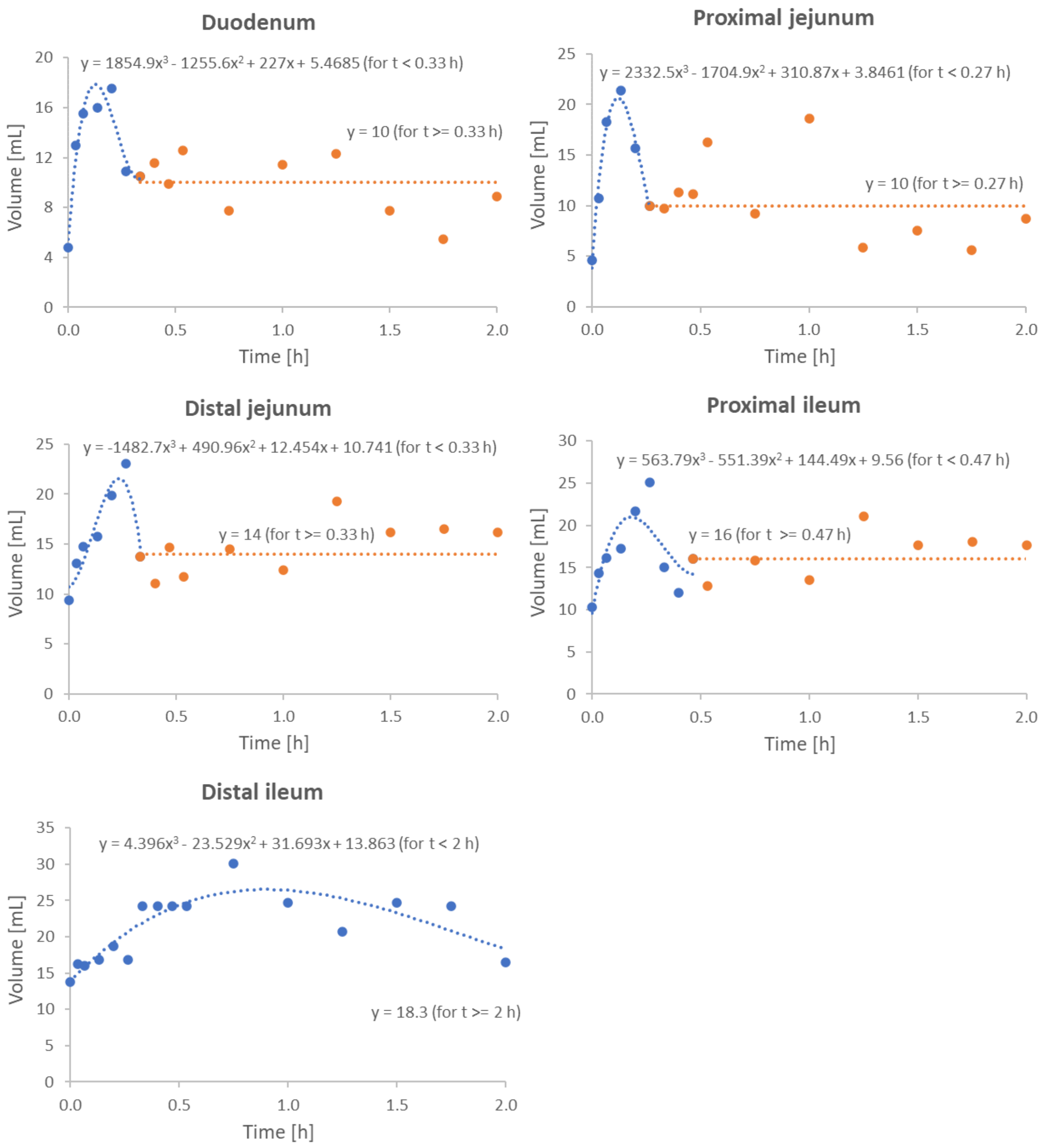


Figure S1. Small intestinal fluid volumes from healthy adult volunteers (blue and orange circles) fitted with the cubic equations in the initial phase (blue dotted line) and the average value in the later phase (orange dotted line).

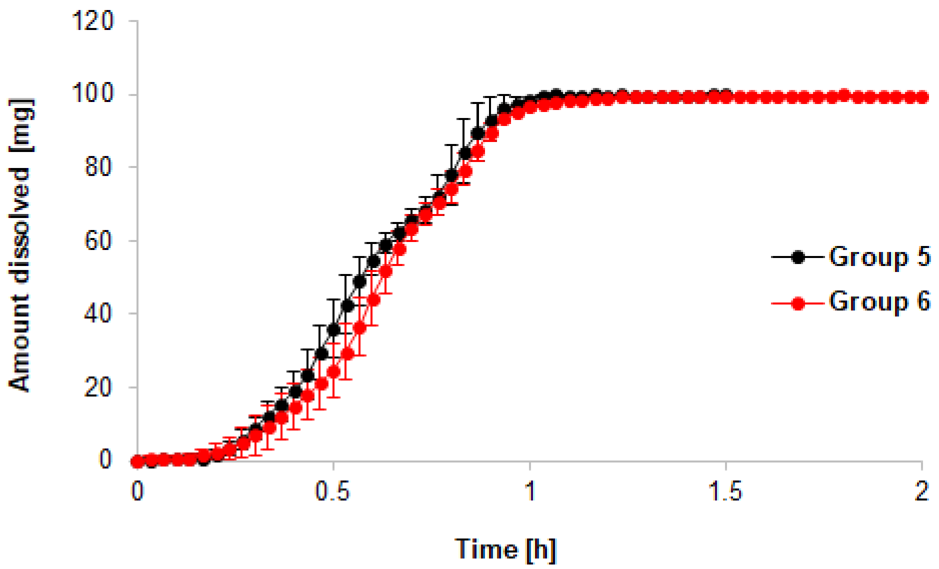

Figure S2. Dissolved amounts of API in the Group 5 and 6 dissolution tests performed for the capsules in *PhysioCell*. The data are presented as means (dots) with standard deviation (bars).

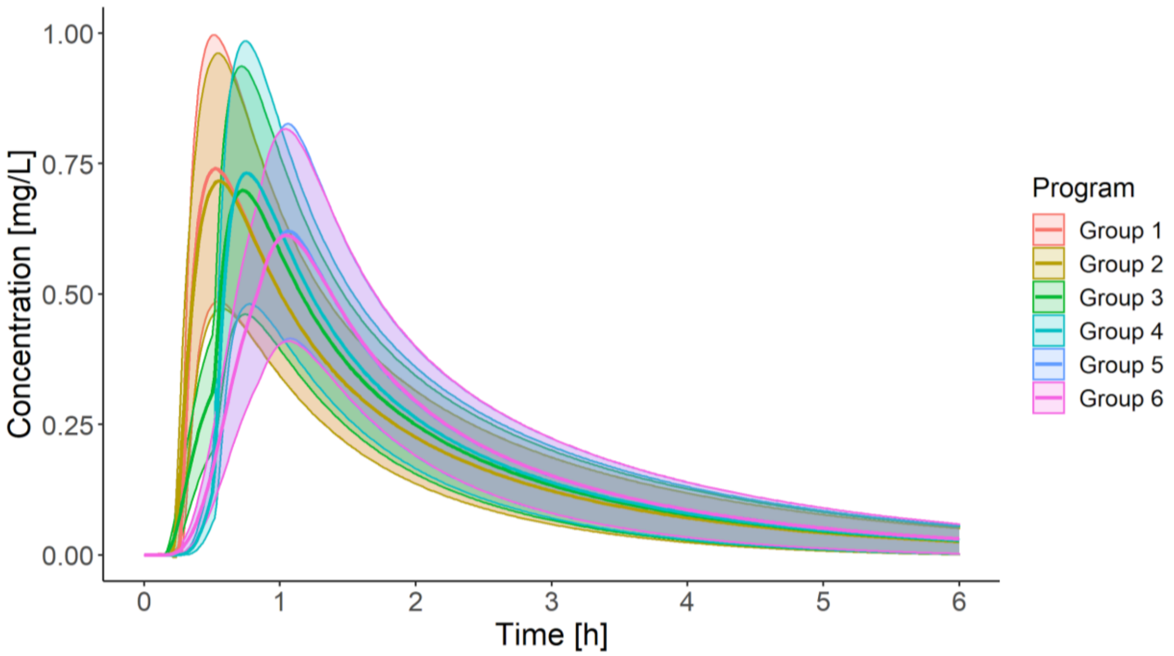

Figure S3. The comparison of the simulated capsule PK profiles for all the tested dissolution programs, assuming fast gastric emptying ($k_{GE}$ = 14 1/h), n = 12. Data are presented as means with SD as ribbons.

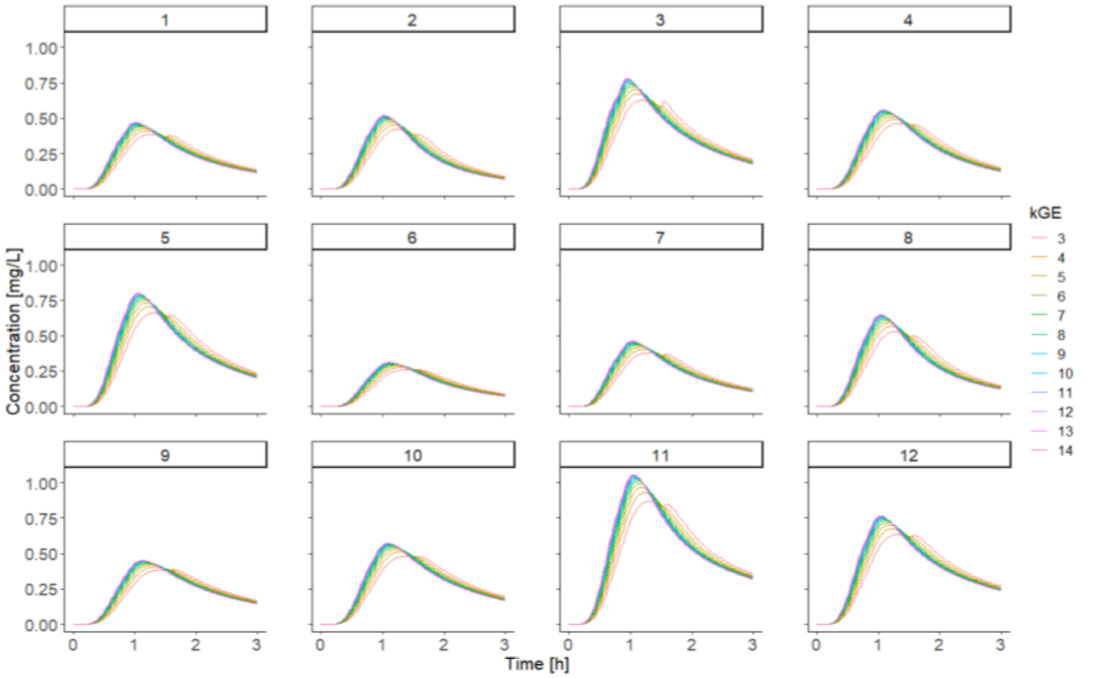


Figure S4. A comparative analysis of the $k_{GE}$ influence on the predicted individual PK profiles for 12 virtual subjects representing Group 6.

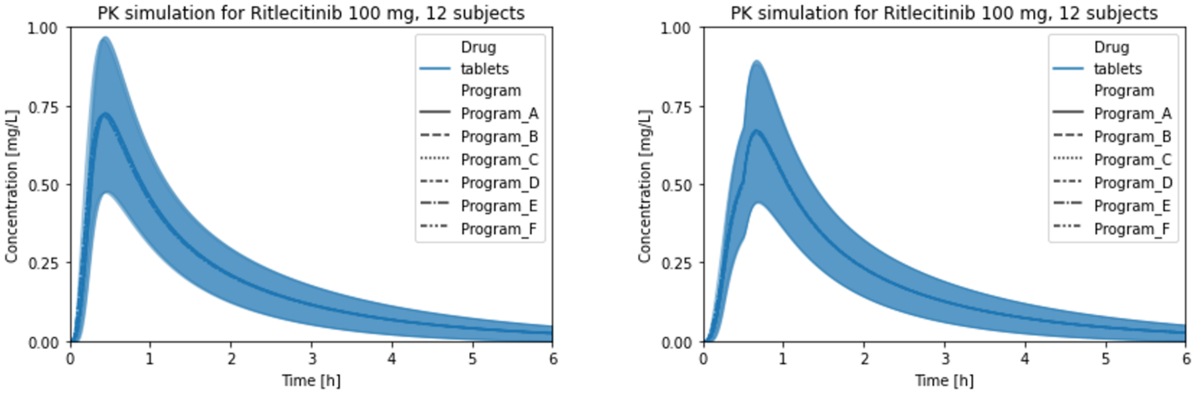


Figure S5. PK profiles (mean ± 1 SD) simulated for 12 virtual subjects after the tablet administration (2 x 50 mg). The simulation was performed for the $k_{GE}$ 14 1/h (left panel) and $k_{GE}$ 3 1/h (right panel). In all the dissolution programs A – F, the GET was 30 min. A single 300 mbar intragastric stress was simulated at 15 min (B and E) or 10 min (C and F) or was absent (A). The constant fluid flow rate of 8 mL/min was applied in A – D, and the gradient flow rate from 50 to 8 mL/min was applied in E and F.
